# Association of *CDKN2A/B* mutations, PD-1, and PD-L1 with the risk of acute lymphoblastic leukemia in children

**DOI:** 10.1007/s00432-023-04974-x

**Published:** 2023-06-14

**Authors:** Yang Ruan, Longlong Xie, Aijun Zou

**Affiliations:** 1 Department of Laboratory Medicine, Hunan Children’s Hospital, Changsha, 410007 China; 2 Pediatrics Research Institute of Hunan Province, Hunan Children’s Hospital, Changsha, 410007 China

**Keywords:** Cyclin dependent kinase inhibitor 2A/B, Programmed cell death protein 1, Programmed cell death ligand 1, Acute lymphoblastic leukemia, Children

## Abstract

**Purpose:**

Currently, the significance of *CDKN2A/B* mutations in the pathogenesis and prognosis of acute lymphoblastic leukemia (ALL) is inconclusive. In this study, we analyzed the genetic and clinical features of children with *CDKN2A/B* mutations in ALL. In addition, we evaluated the expression and significance of programmed cell death protein 1 (PD-1) and programmed cell death ligand 1 (PD-L1) in serum and explored their role in the susceptibility of childhood ALL.

**Methods:**

We sequenced *CDKN2A/B* in the peripheral blood of 120 children with ALL and 100 healthy children with physical examination. The levels of CD4^+^ T, CD8^+^ T, and NK cells were measured by flow cytometry (FCM). Furthermore, the expression of PD-1 and PD-L1 was detected by ELISA.

**Results:**

We found 32 cases of *CDKN2A* rs3088440 and 11 of *CDKN2B* rs2069426 in 120 ALL children. Children with ALL in the *CDKN2A* rs3088440 were more likely to have hepatosplenomegaly (*P* = 0.019) and high risk (*P* = 0.014) than the wild group. In contrast, *CDKN2B* rs2069426 was more likely to develop lymph node metastasis (*P* = 0.017). The level of PD-L1 in the serum of ALL children was significantly higher than that of the control group, and there was no significant difference in PD-1 (*P* < 0.001). Additionally, children with *CDKN2A* rs3088440 had reduced CD8^+^ T cell counts than the wild group (*P* = 0.039).

**Conclusion:**

*CDKN2A* rs3088440 and *CDKN2B* rs2069426 may be related to the occurrence and development of ALL in Chinese children. Additionally, PD-1/PD-L1 may be involved in the immune escape process of ALL, which is expected to become a new target for the treatment of the disease.

## Introduction

Leukemia is a malignant tumor of the hematopoietic system and a serious disease that threatens the life and health of children. According to WHO statistics, malignant tumors have become the second leading cause of death in children after accidental injuries (Young et al. 1986). Acute lymphoblastic leukemia (ALL) is children's most common malignant tumor, accounting for about 80% of ALL children (Shen et al. 2018). Although the treatment of ALL has matured, and its cure rate has risen to 90%, there are still 10% of patients with relapse and poor prognosis following treatment (Karol and Pui 2020). Due to the high incidence of ALL, relapsed or refractory ALL remains the leading cause of tumor-related death in children (Imai 2017). However, the etiology and pathogenesis of ALL are not fully understood. From what is suggested, it may be associated with genetic mutations, viral infections, physical and chemical factors (Bardsiri et al. 2022; Deng et al. 2022; Onyije et al. 2022). Therefore, it is vital to discover the key genes of ALL, biomarkers of ALL malignant proliferation, and to explore the molecular mechanisms of ALL occurrence. Notably, these investigations are valuable in basic research, clinical diagnosis, and treatment.

Genetic and epigenetic mechanisms such as gene mutations, deletions, and DNA methylation can activate proto-oncogenes. Activation of proto-oncogenes, abnormal expression of antioncogene, apoptosis suppressor genes, and abnormalities in cell cycle regulation play essential roles in the development of malignant tumors (Chen et al. 2017; Matthews et al. 2022).

Genome-wide association studies (GWAS) have found that single nucleotide polymorphisms (SNPs) in essential genes are associated with a high risk of ALL. These findings suggest that ALL may be related to polygenic susceptibility (Vijayakrishnan et al. 2018; Pui et al. 2019). However, these studies have not reached a consensus in the worldwide population. Another factor associated with the onset of ALL is the ethnic characteristics of different populations (Hsu et al. 2016). Importantly, several studies involving the genomes of large numbers of patients have suggested that genetic variants may be risk factors for ALL in populations with different patterns of ethnic composition (Fernandes et al. 2022; Yamada et al. 2017).

Cyclin-dependent kinase inhibitor 2A/B (*CDKN2A/B)* is an important regulator of cell growth regulation and apoptosis, while the absence of cell proliferation regulation and cell cycle regulation is crucial for the development and progression of cancer (Hayward et al. 2017; Kathiravan et al. 2019). Notably, it has been suggested that coding variants of *CDKN2A/B* may be associated with susceptibility to ALL in children (Xu et al. 2015). In addition, programmed cell death ligand 1 (PD-L1) is highly expressed in some tumor cells, which is a significant factor in promoting tumor immune escape (Qin et al. 2015; Zhou et al. 2017). PD-1/PD-L1 signaling pathway plays a vital role in tumor immune escape. Furthermore, PD-L1, which is highly expressed on the surface of tumor cells, can inhibit the anti-tumor immune response of T cells, thus allowing tumor cells to evade immune surveillance (Yu et al. 2013).

Currently, because the significance of *CDKN2A/B* gene mutations in the pathogenesis and prognosis of ALL is inconclusive, some of the literature has yielded studies with widely varying results and different conclusions. Therefore, further studies are needed to understand whether *CDKN2A/B* gene mutations, programmed cell death protein 1 (PD-1), and PD-L1, are related to ALL occurrences in children. This study sequenced the *CDKN2A/B* gene in 120 children with ALL. Importantly, we analyzed the expression and significance of PD-1 and PD-L1 in serum and explored their role in the susceptibility of childhood ALL.

## Materials and methods

### Research subjects

A total of 120 children with ALL were admitted to Hunan Children's Hospital selected. ALL included children with blood and bone marrow changes that met the FAB Collaborative Group's diagnostic criteria for ALL (Eys et al. 1986). We excluded other children with non-acute lymphoblastic leukemia and those with genetic disorders. The 100 children in the control group were randomly selected from healthy examinations of the same period. Children with malignancy, hematologic disorders, or family history cannot be included. This study has been approved by the hospital ethics committee (No. HCHLL-2020 -32).

### Research methods

#### *CDKN2A/B* gene mutation detection

Venous whole blood samples were collected using EDTA tubes. Plasma was separated by centrifugation (1,000 rpm, 15 min) within 2 h after blood collection and then stored at – 80 ℃ until the analysis. The DNA extraction kit (Tiangen Biotech Corp) was used to extract whole blood specimen DNA. The exon coding sequence of the *CDKN2A/B* gene was determined according to the database, primers were designed, and the extracted DNA was sequenced by Sanger. Bioinformatics analysis was performed after sequencing to determine the mutation site of the *CDKN2A/B* gene.

#### Detection of PD-1, PD-L1

Plasma levels of soluble PD-1 and PD-L1 were assayed using enzyme-linked immunosorbent assay (ELISA) kits SEA751Hu and SEA788Hu (Cloud-Clone Corp, TX, USA). Their assay range was 0.156–10 ng/mL with detection limits of 0.063 ng/mL and 0.056 ng/mL, respectively. PD-1 and PD-L1 were tested in strict accordance with the kit instructions.

#### Detection of CD4^+^T cells, CD8^+^ T cells, and NK cells

The lymphocyte subsets were identified and determined using the BD FACSCanto™II Flow Cytometer. For the flow cytometry analyses, the reagent cocktail (20 μL) containing CD3 FITC, CD4 PE-Cy7, CD8 APC-Cy7, CD16 PE, CD19 APC, CD45 PerCP-Cy5.5, and CD56 PE was added to 50 μL whole blood. All of these were purchased from BD Pharmingen. For each sample, at least 10,000 cells were analyzed and the percentage of the cells expressing CD4^+^ T cells (CD45^+^CD3^+^CD4^+^), CD8^+^ T cells (CD45^+^CD3^+^CD8^+^), NK cells (CD45^+^CD16^+^ CD56^+^), and B cells (CD45^+^CD19^+^) markers were evaluated.

### Statistics

SPSS 20.0 software was used for the statistical analysis of the data. Continuous variables conforming to normal distribution were expressed as mean ± standard deviation ($\overline{x}$ ± s). Enumeration data were expressed as rate and percentage. The Chi-square test (χ^2^) was used to compare the data between the two groups. The odds ratio (OR) and 95% confidence interval (CI) were used to represent the degree of association. Multiple related factors were analyzed using Logistic regression. A significant difference was considered when the p-value < 0.05.

## Results

### Correlation analysis of *CDKN2A/B* gene mutation with childhood ALL

We found a mutation site rs3088440 in the 3'-UTR of exon 3 of *CDKN2A*. Additionally, rs3088440 (C > T) was found in 32 of 120 ALL specimens (26.7%) in this study. In the control group, 13 of 100 specimens (13.0%) had rs3088440 (C > T). Our statistical analysis showed that the mutation rate of rs3088440 (C > T) in the ALL group was higher than in the control group and that the difference was statistically significant (*P* = 0.012, OR = 2.43, 95%CI: 1.197–4.947) (Table 1).

**Table 1:** *CDKN2A* and *CDKN2B* genotypes in the ALL and control groups

SNP	ALL (%)	Controls (%)	$\chi^{2}$	*P*	OR(95%CI)
*CDKN2A* rs3088440 (C > T)	32(26.7%)	13(13.0%)	6.262	**0.012**	2.43(1.197–4.947)
*CDKN2A* rs3088440 wild type	88(73.3%)	87(87.0%)			
*CDKN2B* rs2069426 (C > A)	11(9.2%)	2(2.0%)	5.039	**0.025**	4.95(1.070–22.863)
*CDKN2B* rs2069426 wild type	109(90.8%)	98(98.0%)			

*SNP* single nucleotide polymorphism, *OR* odds ratio, *CI* confidence interval

We also found a mutation site rs2069426 (C > A) in the non-exon sequence of *CDKN2B*. Rs2069426 (C > A) was found in 11 of 120 ALL specimens (9.2%) in this study and 2 of 100 specimens (2.0%) in the control group (*P* = 0.025, OR = 4.95, 95% CI: 1.070–22.863) (Table 1).

### Clinical characteristics of patients with ALL and controls

The age of onset, white blood cell count, lymph node metastasis, and hepatosplenomegaly were significantly different in ALL children compared with the control group (*p* < 0.05) (Table 2). The children with *CDKN2A* rs3088440 mutations were more likely to develop hepatosplenomegaly and be at higher risk than the wild group. However, children with *CDKN2B* rs2069426 mutations were more likely to have lymph node metastasis (Table 3).

**Table 2:** Clinical characteristics of ALL and control patients

Clinical characteristics	n	n(%)		$\chi^{2}$	*p*
		ALL patients	Controls		
*Gender*					
Males	139	76(63.3)	63(63.0)		
Females	81	44(36.7)	37(37.0)	0.003	0.959
*Age*					
< 5 years	106	67(55.8)	39(39.0)		
≥ 5 years	114	53(44.2)	61(61.0)	6.191	**0.013**
*White blood cell count*					
< 50 × 10^9^/L	198	98(81.7)	100(100.0)		
≥ 50 × 10^9^/L	22	22(18.3)	0	20.370	**< 0.001**
*Lymphatic metastasis*					
Yes	47	47(39.2)	0		
No	173	73(60.8)	100(100.0)	49.807	**< 0.001**
*Hepatosplenomegaly*					
Yes	77	77(64.2)	0		
No	143	43(35.8)	100(100.0)	98.718	**< 0.001**

**Table 3:** Relationship between *CDKN2A* and *CDKN2B* gene status, and clinical characteristics in children with ALL

Clinical characteristics	n	*CDKN2A* rs3088440 n (%)		*p*	n	*CDKN2B* rs2069426 n (%)		*p*
		Mutation	Wild type			Mutation	Wild type	
*Gender*								
Males	76	18(56.3)	58(65.9)		76	9(81.8)	67(61.5)	
Females	44	14(43.7)	30(34.1)	0.332	44	2(18.2)	42(38.5)	0.182
*Age*								
< 5 years	67	19(59.4)	48(54.5)		67	7(63.6)	60(55.0)	
≥ 5 years	53	13(40.6)	40(45.5)	0.638	53	4(36.4)	49(45.0)	0.585
*White blood cell count*								
< 50 × 10^9^/L	98	25(78.1)	73(83.0)		98	10(90.9)	88(80.7)	
≥ 50 × 10^9^/L	22	7(21.9)	15(17.0)	0.545	22	1(9.1)	21(19.3)	0.406
*Lymphatic metastasis*								
Yes	47	11(34.4)	36(40.9)		47	8(72.7)	39(35.8)	
No	73	21(65.6)	52(59.1)	0.517	73	3(27.3)	70(64.2)	**0.017**
*Hepatosplenomegaly*								
Yes	77	26(81.2)	51(58.0)		77	7(63.6)	70(64.2)	
No	43	6(18.8)	37(42.0)	**0.019**	43	4(36.4)	39(35.8)	0.969
*Risk* *stratification*								
Low-risk	58	12(37.5)	46(52.3)	0.284	58	7(63.6)	51(46.8)	0.342
Middle-risk	58	17(53.1)	41(46.6)	0.059	58	4(36.4)	54(49.5)	0.587
High-risk	4	3(9.4)	1(1.1)	**0.014**	4	0	4(3.7)	0.461

### Comparison of PD-1, PD-L1, CD4^+^, CD8^+^ and NK between ALL children and controls

PD-L1 was significantly higher in the serum of children with ALL than in the control group. In contrast, PD-1 was not significantly different. CD4^+^ and CD8^+^ T cells were significantly lower in children with ALL than in controls, and there was no significant difference in NK cells (Table 4). Children with *CDKN2A* rs3088440 had lower CD8^+^ T cells than the wild-type group. However, there was no significant difference between *CDKN2B* rs2069426 and wild type group (Table 5).

**Table 4:** Comparison of PD-1, PD-L1, CD4^+^, CD8^+^, NK cells between ALL children and control group (pg/ml, $\overline{x} \pm s$)

Group	PD-1	PD-L1	CD4^+^	CD8^+^	NK
ALL	1.13 ± 6.05	406.27 ± 1034.73	26.13 ± 13.61	20.20 ± 11.84	13.64 ± 16.21
Control	0.30 ± 2.96	36.67 ± 126.78	35.90 ± 6.64	23.73 ± 5.17	11.50 ± 5.47
*t*	1.323	3.878	− 6.936	− 2.945	1.356
*P*	0.188	**< 0.001**	**< 0.001**	**0.004**	0.177

**Table 5:** Comparison of PD-1, PD-L1, CD4^+^, CD8^+^, NK cells between *CDKN2A* rs3088440, *CDKN2B* rs2069426 and wild type group (pg/ml, $\overline{x} \pm s$)

	*CDKN2A* rs3088440		*p*	*CDKN2B* rs2069426		*p*
	Mutation	Wild type		Mutation	Wild type	
PD-1	3.18 ± 10.99	0.38 ± 2.22	0.162	0 ± 0	1.24 ± 6.34	0.52
PD-L1	415.08 ± 809.98	403.07 ± 1109.35	0.955	237.84 ± 599.37	423.27 ± 1069.24	0.573
CD4^+^	25.15 ± 16.69	26.51 ± 12.42	0.675	26.33 ± 10.99	26.13 ± 13.90	0.963
CD8^+^	16.51 ± 10.32	21.55 ± 12.12	**0.039**	20.95 ± 8.43	20.13 ± 12.16	0.827
NK	12.61 ± 16.14	14.01 ± 16.31	0.677	19.15 ± 24.05	13.08 ± 15.25	0.238

### Logistic regression analysis

Logistic regression analysis showed that age, *CDKN2A* rs3088440, *CDKN2B* rs2069426, and PD-L1 were independent risk factors for ALL (Table 6).

**Table 6:** Logistic regression analysis of the relationship between clinicopathological features and ALL

Factors	B	SE	Wald	*P*	OR(95%CI)
X1 (gender)	− 0.061	0.302	0.041	0.839	0.940(0.520–1.700)
X2 (age)	− 0.658	0.291	5.105	**0.024**	0.518(0.292–0.916)
X3 (rs3088440)	0.920	0.379	5.899	**0.015**	2.509(1.194–5.272)
X4 (rs2069426)	1.600	0.796	4.044	**0.044**	4.955(1.041–23.572)
X5 (PD-L1)	0.783	0.253	9.612	**0.002**	2.189(1.334–3.591)
Constant	0.094	0.293	0.104	0.747	1.099

*SE* standard error, *OR* odds ratio, *CI* confidence interval

## Discussion

The cyclin-dependent kinase inhibitor 2A/B (*CDKN2A/B*) gene is located in the chromosome 9p21 region. It is a vital cancer suppressor gene and belongs to the family of cell cycle-dependent enzyme inhibitor genes. *CDKN2A* encodes the cyclin-dependent kinase inhibitors p16^INK4A^ and replacement reading frame protein p14^ARF^, while *CDKN2B* encodes the p15^INK4B^ (Krieger et al. 2010). These three proteins, as components of the RB1 and TP53 pathways, negatively regulate the G1-S transition in the cell cycle during proliferation to maintain normal cell growth (Salas et al. 2016). p16^INK4A^ and cyclin competitively bind to cell cyclin-dependent protein kinase (CDK4/6) and inhibit CDK4/6 kinase activity. In doing so, they render retinoblastoma protein (pRb) unphosphorylated. Notably, these steps prevent cells from entering the S phase and initiating DNA synthesis, inhibiting cell proliferation. At the same time, highly phosphorylated pRb can induce the expression of p16^INK4A^, which in turn can inhibit the phosphorylation of Rb protein (Zhao et al. 2016). Thus, p16 plays a negative feedback regulatory role in the cell cycle regulatory pathway p16^INK4A^-CDK4/6-pRb-E2F. Its abnormality can cause uncontrolled cell proliferation, leading to tumors (Zhao et al. 2016). Therefore, the inactivation of *CDKN2A/B* may lead to rapid or uncontrolled cell growth and even cancer formation. Inactivation of *CDKN2A/B* occurs through mutation, deletion, or methylation (Sulong et al. 2009). Furthermore, mutations or deletions in the *CDKN2A/B* gene are associated with various tumors, including breast cancer, melanoma, ovarian cancer, and lung adenocarcinoma (ShahidSales et al. 2018; Chan et al. 2017; Reinhardt et al. 2018; Cocco et al. 2017; Jiang et al. 2016; Mullighan and Downing 2009).

ALL is the most common malignancy in children (Terwilliger and Hay 2017). Studies have shown that specific gene mutations may be associated with abnormal signaling pathways, and their abnormal activation can promote oncogenic changes in ALL (Burns et al. 2018). Additionally, genetic variation is strongly associated with susceptibility to ALL in children (Li et al. 2022). Alteration of the *CDKN2A/B* locus is one of the hallmarks of ALL (Celia et al. 2021). *CDKN2A* exon variants are associated with susceptibility to childhood ALL (Vijayakrishnan et al. 2017; Walsh et al. 2015). The absence of *CDKN2A/B* may indicate poor prognosis in adult and pediatric ALL patients, which may be related to the pathogenesis of the disease (Ribera et al. 2017; Qian et al. 2019; Braun et al. 2017; Zhang et al. 2019; Kumari et al. 2022). However, some scholars believe that there is no correlation (Zutven et al. 2005; Mirebeau et al. 2006). These differences in study results may be due to different clinical characteristics, including race, age, and subtype (Liao et al. 2016; Guo et al. 2014; Prasad et al. 2010). Xu et al. (2015) found that the *CDKN2A* gene was related to the occurrence of ALL in children, and it could increase the probability of leukemia transformation. Additionally, Maude et al. (2015) performed sequencing analysis on 204 children with ALL chemotherapy and found that more than 90% of children with recurrent ALL had *CDKN2A* gene deletions. Likewise, the *CDKN2A* SNP (rs3731249) is associated with susceptibility to ALL (Walsh et al. 2015; Vijayakrishnan et al. 2015; Gutierrez-Camino et al. 2017). A meta-analysis with a large sample size showed that SNPs at *CDKN2A* (rs3731217 and rs3731249) were significantly associated with the risk of ALL. Individuals carrying these two SNP risk alleles had 0.72-fold and 2.26-fold increased disease susceptibility, respectively (Zhou et al. 2018). Moreso, rs3731249 and rs2811709 have been linked to B-ALL susceptibility in Spaniards (Gutierrez-Camino et al. 2017). Furthermore, rs3731246 can be used as a risk marker for ALL susceptibility in Yemeni children (Al-Absi et al. 2017). In addition to germline mutations in the *CDKN2A* exon, SNPs predisposed to B-ALL have been identified in introns and noncoding regions such as promoters (Sherborne et al. 2010). Notably, these SNPs regulate *CDKN2A* gene expression (Hungate et al. 2016). In particular, many loci associated with susceptibility to ALL were found in noncoding genome regions. Importantly, non-coding elements have a relevant role in cancer development (Khurana et al. 2016). Mutations in the *CDKN2A* locus can modify the protein interaction domain in INK4a. It can affect the interaction with other proteins, such as MYB, or lead to the mislocalization of INK4a protein in the nucleus (Healy et al. 2007; Britigan et al. 2014). Hungate et al (2016) also found through functional analysis that rs662463 could regulate *CDKN2B* expression through CEBPB signal and affect the risk of B-ALL in children. Therefore, mutations in *CDKN2A* (− 222 T>A) and *CDKN2B* (593A>T, C) may play a role in susceptibility to childhood leukemia (Healy et al. 2007). Collectively, these studies suggest that the *CDKN2A/B* gene may play a crucial role in the development of ALL.

We performed Sanger sequencing to detect *CDKN2A/B* gene mutations in the blood of 120 children with ALL and 100 healthy children with a physical examination. These investigations found a missense mutation rs3088440 in exon 3 of *CDKN2A. CDKN2B* has a non-exon mutation rs2069426. Likewise, the age of onset, white blood cell count, lymph node metastasis, and hepatosplenomegaly was significantly different in the ALL group compared with the control group (*p* < 0.05). Children with *CDKN2A* rs3088440 mutation were more likely to develop hepatosplenomegaly and high risk than the wild group. Additionally, children with *CDKN2B* rs2069426 mutation were more likely to have lymph node metastasis. Some studies have shown that the risk allele of SNPs in the 3′-UTR of *CDKN2A* may alter the binding capacity of transcription factors, thereby affecting the expression of target genes (Hesari et al. 2019). Furthermore, we identified a polymorphic locus rs3088440 in the 3'-UTR of *CDKN2A* in children with ALL. The 3′-UTR region is a transcriptional regulatory region, and SNPs can affect mRNA stability and protein synthesis by regulating regulatory elements in the region (Dong et al. 2017). As previously demonstrated, the C allele in the rs3088440 variant facilitates the binding of c-Myb to the *CDKN2A* transcriptional regulatory region, which may lead to the repression of the *CDKN2A* gene and impair its normal function in cell cycle regulation (Stenman et al. 2010). Moreover, rs3088440 was positively correlated with ALL mortality, and the higher the frequency of this variant allele, the higher the incidence and mortality of ALL (Fernandes et al. 2022). Therefore, *CDKN2A* rs3088440 may be associated with the development of childhood ALL. Our study showed that there is a mutation site rs2069426 on the *CDKN2B*. This missense mutation can turn leucine into isoleucine. This mutation may alter the cis-regulatory splicing element and thus affect *CDKN2B* expression to function (Burd et al. 2010). Therefore, these results suggest that *CDKN2A* rs3088440 and *CDKN2B* rs2069426 may be associated with the susceptibility to childhood ALL.

The environment of tumor survival is also known as the tumor microenvironment. It contains various types of cells, such as tumor, stromal, and immune cells (Hui and Chen 2015). Immune cells include T cells, B cells, macrophages, and other key cells. Various components in the microenvironment are in constant communication with tumor cells, so the tumor microenvironment plays a significant role in the occurrence and progression of tumors (Qin et al. 2017). CD8^+^ T cells play a major role in killing tumor cells in the tumor microenvironment (Farhood et al. 2019). Many studies have found that the function of T cells in the tumor microenvironment is inhibited and that the proliferation ability is limited and reaches the exhaustion state, eventually leading to the proliferation and metastasis of tumor cells (Majzner and Mackall 2019; Minton 2020; Zhao et al. 2020). Notably, PD-1 and PD-L1 interactions provide an immunosuppressive environment for tumor growth. Studies have shown that PD-1 levels are increased in pancreatic cancer, non-small cell lung cancer, nasopharyngeal cancer, chronic lymphocytic leukemia, advanced rectal cancer, and other cancers (Kruger et al. 2017; Bian et al. 2019; Hejleh et al. 2019; Chang et al. 2019; Meyo et al. 2020; Ruan et al. 2019; Tominaga et al. 2019). Additionally, PD-1/PD-L1 expression is higher in solid and leukemic tumors and has been an independent predictor of survival in some experiments (Kiyasu et al. 2015; Miyoshi et al. 2016). Consequently, elevated plasma PD-L1 levels are associated with advanced disease, clinical stage, and poor prognosis in cancer patients (Bian et al. 2019; Khan et al. 2020). The interaction of PD-1 and PD-L1 can inhibit T cell effector functions, such as cytotoxicity and cytokine release, limit the proliferation and survival of T cells, and induce apoptosis of tumor-specific T cells (Freeman et al. 2000). In addition, CD4^+^ T cells differentiate into Fox3^+^ regulatory T cells (Wang et al. 2008). These co-inhibitory pathways are critical mechanisms of tumor immune escape. Furthermore, blocking this pathway can improve T-cell function and the survival of cancer patients (Zitvogel and Kroemer 2012; Iwai et al. 2017).

Our study found that the serum PD-L1 was significantly higher in children with ALL than in the control group, and CD4^+^ and CD8^+^ T lymphocytes were lower. Importantly, *CDKN2A* rs3088440 had lower CD8^+^ T lymphocytes than the wild group. Moreso, the logistic regression analysis showed that *CDKN2A* rs3088440, *CDKN2B* rs2069426, PD-L1, and age were independent risk factors for childhood ALL. Studies have found that the binding of PD-L1 to its receptor PD-1 results in the phosphorylation of immunoreceptor tyrosine-based inhibitory motif (ITIM) and immunoreceptor tyrosine transforming motif (ITSM) in the cytosolic domain of PD-1 to be phosphorylated by Src family tyrosine kinases. Further recruitment of Src homology 2 domain-containing phosphatase (SHP) to phosphorylated tyrosine residues inactivates T cells to activate essential cytokines and proteins, ultimately leading to functional inhibition of T cells (Shimizu et al. 2020; Ok and Young 2017). PD-L1 is mainly released by tumor cells and mature dendritic cells and can induce apoptosis of CD4^+^ and CD8^+^ T lymphocytes (Frigola et al. 2012). PD-L1 also protects tumor cells from the cytotoxic effects of type I and type II interferons and cytotoxic T lymphocyte-mediated cytolysis (Gato-Canas et al. 2017). Therefore, PD-L1 and PD-1 are vital players in the tumor microenvironment and represent therapeutic targets against tumors. Studies have shown that CDK4 functions as a negative regulator of PD-L1 expression by indirectly regulating its ubiquitination (Zhang et al. 2018). Thus, we speculated that *CDKN2A* rs3088440 might up-regulate the expression of PD-L1 through CDK4, hence inducing CD8^+^ T lymphocytes depletion and ultimately leading to tumor cell proliferation and metastasis.

In conclusion, *CDKN2A* rs3088440 and *CDKN2B* rs2069426 may be related to the occurrence and development of ALL in Chinese children. Additionally, PD-1/PD-L1 may be involved in the immune escape process of ALL, which is expected to become a new target for the treatment of the disease. Therefore, the detection and localization of *CDKN2A/B* gene mutation and PD-L1 may provide a reasonable basis for the targeted treatment strategy of ALL.

## Data Availability

The data presented in this study are available from the corresponding author upon request.
